# Advancing use of long‐acting and extended delivery HIV prevention and treatment regimens

**DOI:** 10.1002/jia2.26126

**Published:** 2023-07-13

**Authors:** Sinéad Delany‐Moretlwe, Charles Flexner, José A. Bauermeister

**Affiliations:** ^1^ Wits Reproductive Health and HIV Institute University of the Witwatersrand Johannesburg South Africa; ^2^ Divisions of Clinical Pharmacology and Infectious Diseases Johns Hopkins University Baltimore Maryland USA; ^3^ Department of Family & Community Health University of Pennsylvania Philadelphia Pennsylvania USA

Long‐acting and extended delivery (LAED) HIV treatment regimens are becoming increasingly accessible to consumers. After more than a decade of clinical research, long‐acting antiretroviral formulations have been approved for treatment and prevention [1, 2]. Long‐acting injectable cabotegravir (CAB)‐rilpivirine has begun to receive regulatory approval for HIV treatment across the globe, including the United States, the UK, Europe, Australia and Canada. Similarly, new LAED regimens are securing regulatory approval for HIV prevention. A monthly dapivirine vaginal ring (DVR) for women at high risk for HIV acquisition has received regulatory approval in several African countries, a positive opinion from the European Medicines Agency, and is under consideration by other regulatory agencies. Following the successful HPTN 083 and 084 trials, the U.S. Food and Drug Administration approved a bimonthly injection of long‐acting injectable CAB for Pre‐Exposure Prophylaxis (PrEP) in 2021 [3], with subsequent approvals being secured or pursued across other countries. The United States Agency for International Development is supporting large demonstration projects for both the DVR and CAB to facilitate rollout in multiple sub‐Saharan African countries. These strategies represent the forefront of numerous new LAED product candidates and delivery strategies now advancing in early‐stage development or clinical trials.

LAED regimens have the potential to reshape the HIV treatment and prevention landscape through dosing schedules that are measured in months rather than days. To realize their maximum impact, it is important to recognize that product innovations in HIV and other areas of medicine face a variety of challenges when moving to wide‐scale implementation [4, 5]. An instructive example is found in oral PrEP [6, 7], where meaningful access and use have lagged regulatory approvals in many settings due to patient, provider and healthcare system factors. Steep inequities in uptake and early discontinuation continue to limit the global public health impact of oral PrEP.

There are questions about whether future LAED regimens could face similar challenges in their delivery, uptake and persistent use, as well as concerns about facilitating access in resource‐constrained environments and in key populations. LAED regimens have the potential to overcome many of these challenges, but questions remain about how best to offer HIV prevention and treatment choices, how to manage product switching and what constitutes optimal service delivery while using LAED products. For this supplement, we invited scholars to submit multidisciplinary articles designed to advance the development, future use and equitable delivery of LAED regimens for HIV prevention and treatment. We received several abstracts spanning original research, commentaries, reviews and viewpoints. After careful review and deliberation, the editorial team selected 16 contributions that illustrate current LAED advances and challenges to improve the development, retention and equitable delivery of LAED regimens across low‐, middle‐ and high‐income countries.

Users’ desires and choices may also impact LAED uptake and persistent use. As illustrated by the studies included in the first section of our supplement, a deeper understanding of end‐users’ preferences can guide the development of new LAED and service delivery models and emphasize their significance in creating demand for LAED products.

Graham et al.’s [8] study examined the preferences for potential long‐acting antiretroviral therapies (LA‐ART) regimens among people with HIV in the United States. Using a discrete choice experiment, they found that LA oral tablets were the only mode preferred over the current daily oral treatment. Annual implants and injections were the next most preferred LA‐ART options. Longer time between doses was preferred, and administration at home was preferred to clinics, which were preferred to pharmacies. Future research should investigate the sources of preference heterogeneity and actual uptake of and adherence to LA‐ART products when available.

Biello et al.’s [9] research article focused on the correlates of preferences for next‐generation HIV prevention products among a national U.S. sample of young men who have sex with men (YMSM). Given the low uptake of daily oral PrEP, it is essential to understand end‐users’ preferences and concerns about PrEP products to ensure high acceptability and penetration. In a series of conjoint analyses, participants prioritized efficacy, absence of side effects and costs when considering different PrEP products. Interestingly, their analyses found that age, insurance status, sexual behaviour, PrEP use history, HIV and Sexually Transmitted Infections (STI) testing history, and STI diagnoses influenced YMSM's decisions to prioritize next‐generation modalities over daily oral PrEP.

Finally, Lorenzetti et al.’s [10] review examined the values and preferences regarding the use of injectable PrEP to prevent HIV acquisition. Their review highlighted that there is often a preference for injectable PrEP across populations, but variation within and across groups and regions was also observed. While many stakeholders indicated that injectable PrEP could help address adherence challenges associated with daily or on‐demand dosing for oral PrEP and may be a better lifestyle fit for individuals seeking privacy, discretion and infrequent dosing, end‐users identified concerns related to injectable agents, including fear of needles, injection site pain and concerns about body location for the injections, logistical challenges and waning or incomplete protection.

Taken together, these studies underscore the importance of acknowledging choice as a key tenet of the increasing availability of HIV prevention and care products and ensuring that programmes help address users’ needs and concerns to maximize their appeal and behavioural congruence.

For LAED to achieve its potential impact on the HIV epidemic, it needs to reach populations with the greatest need. These populations often face disparities in health access, either within countries or regions where health resources are limited. Mgodi and colleagues’ commentary [11] focuses on these challenges and provides recommendations to advance the use of LAED for HIV prevention and treatment in sub‐Saharan Africa. In addition to discussing concerns about LAED costs, they highlight the need to train and retain more healthcare providers, implement task shifting, invest in healthcare infrastructure and integrate healthcare services. They call for innovation in laboratory diagnostics and the development of non‐injectable long‐acting formulations for the region.

Recognizing these constraints, four manuscripts focus on cost considerations as a critical barrier to access and discuss strategies that can be leveraged to overcome these challenges. Gaayeb and colleagues [12] discuss voluntary licensing ahead of patent expiry as a strategy to accelerate access to affordable HIV treatment and prevention products. They propose 10 enablers of voluntary licensing of Intellectual Property (IP) rights, with special consideration given to early identification of products designed for low‐ and middle‐income country contexts, technology transfer and innovative partnerships for product development, strategies to optimize regulatory review and de‐risking mechanisms.

Meyer‐Rath et al. [13] extend this focus on cost and access by considering demand and supply‐side factors that can accelerate CAB access in low‐ and middle‐income countries, using South Africa as an example. They argue that guaranteed market volumes based on stimulating and sustaining demand for HIV prevention products like CAB through scaled‐up national programmes can encourage price reductions if coupled with expanded manufacturing capacity.

Jenkins et al. [14] echo many of these sentiments in their roadmap for investment decision‐making and product introduction based on learnings from product introduction models for contraceptives, Tuberculosis (TB) treatment and dolutegravir. They reiterate the importance of partner alignment, the need for advance preparation by national programmes with a focus on policies and guidelines, the importance of community engagement to ensure demand and acceptable service delivery models, the critical role of generic manufacture and expedited regulatory reviews. Castor and colleagues [15] supplement our understanding of cost barriers through a scoping review that analyses PrEP cost study data and identifies gaps in cost information for implementation domains. The authors argue that limited cost information has the potential to undermine product introduction and scale‐up in access. In situations where resources and budgets are constrained, greater consideration may need to be given to budget allocation to priority implementation domains that can yield health system efficiencies.

Warren et al. [16] emphasize the importance of accelerating the design of CAB implementation studies that adequately address priority knowledge gaps. As additional long‐acting HIV prevention products become available over the next 5 years, the delivery of multiple regimens may become more complex. Therefore, expanded stakeholder engagement and ongoing coordination with the WHO will accelerate the adoption of evidence‐based policies and wide‐scale implementation, and lessons from the Biomedical Prevention Implementation Collaborative (BioPIC) may inform strategies to introduce these new long‐acting HIV prevention products across settings. These considerations must also align with a country's HIV epidemiologic profile. Stansfield et al.’s [17] research article highlights how high PrEP coverage with CAB‐LA might substantially reduce HIV in settings that experience high HIV incidence. However, the paper also serves as a reminder that LAED regimens may not be a silver bullet and that careful consideration of the epidemiological context is essential as part of the introduction and scaling of LAED regimens.

Ongoing research and evaluation will help inform best practices as the menu of HIV prevention and care regimens is implemented and scaled. As the final section of our supplement highlights, these efforts will require multisectoral partnerships throughout the LAED development and dissemination pipeline and must ensure that all communities who could benefit from LAED regimens are included and represented.

Kim et al. [18] argue that there is a need for a robust, multidisciplinary research agenda that produces additional long‐acting treatment options, in addition to strategies that support their effective and equitable use. A robust and multilevel LAED research agenda will require multisectoral partnerships to advance innovations in long‐acting regimen development, clinical monitoring, behavioural support interventions and implementation science.

Grimsrud et al.'s [19] commentary reminds us that the development of new LAED should be based on consideration of how people currently receive HIV treatment and prevention services, if we are to achieve improvements in access and outcomes. Future positioning of treatment and prevention LAED is likely to be different. For treatment, LAED regimens may address challenges with adherence, but their delivery should provide clear advantages over existing oral products to be scaled. For prevention, LAED regimens expand a potential PrEP user's choice of methods, but like other methods, they need to be delivered in a manner that allows for re‐initiation for individuals whose risk profiles may vary over time.

Celum et al.’s [20] commentary reinforces the importance of learning lessons from oral PrEP as we consider how to optimize the delivery of long‐acting PrEP methods. Strategies that expand PrEP delivery options, including telePrEP, pharmacy‐based PrEP, key population‐led services and mobile venues, need further evaluation. Integrated delivery models that include STI testing and treatment, contraception for people not desiring to become pregnant, PrEP for pregnant women in high HIV prevalence settings, and gender‐affirming hormones and support for transgender persons are needed. The lessons learned from the delivery of oral PrEP about demand creation, informed client decision‐making, provider training, adherence support and service delivery models are relevant to the delivery of long‐acting PrEP and integration with other services.

Three manuscripts serve as a reminder to ensure that the benefits of LAED accrue to populations that are frequently left behind. Hosek et al.’s [21] viewpoint reminds us that the needs of LAED regimens must be centred around the needs and experiences of adolescents and young adults. Given the persistent disparities in HIV outcomes among youth, the authors renew a call to support the development, effective implementation and equitable delivery of LAED products for HIV prevention and treatment by aligning new technologies with existing youth‐focused programmes. White et al.’s [22] viewpoint also notes the importance of including pregnant and breastfeeding people in trials of novel LAED PrEP agents, and discusses the work needed to overcome community and regulators’ concerns about the inclusion of pregnant women in trials. Success in the development and implementation of LAED requires partnership with communities to build confidence and foster inclusion of populations traditionally regarded as vulnerable. Gandhi et al.’s [23] viewpoint reflects the challenge of evaluating LAED in viraemic patients who experience adherence challenges for social and structural reasons. The authors argue for a new approach to trial design that would allow evaluation of LAED in this population and aid in implementation in real‐world settings.

Taken together, the contributions to this supplement highlight the opportunities and synergize the rollout and scale‐up of LAED regimens across the globe. The manuscripts in this supplement note how structural, policy, community and healthcare contexts might facilitate or hinder the delivery of LAED regimens. Like other prevention and care efforts, the success of LAED regimens will require countries to allocate the appropriate resources required for a tailored strategy that maximizes LAED access and persistent use among its constituents. As illustrated by the manuscripts in this supplement, these tailored efforts will require an understanding of users’ needs across settings, collective buy‐in from stakeholders and communities, and comfort in creating evidence‐based implementation guidelines to support patients’ switches between HIV prevention and care regimens. Moreover, while the existing data indicate the potential of LAED regimens to help achieve global HIV prevention and care goals, continued investments are needed to ensure equity in access among key populations. LAED regimen research with understudied and/or excluded populations should remain a priority to ensure their inclusion in regulatory guidelines. We hope that the key considerations raised in this supplement will serve to advance the field as existing and emerging LAED regimens are approved and implemented.

## COMPETING INTERESTS

The authors declare no competing interests.

## AUTHORS’ CONTRIBUTIONS

JAB and SD‐M contributed to the initial draft of the manuscript. CF provided feedback, reviewed and edited the draft, and approved the final version prior to submission.

## FUNDING

Publication of this open‐access supplement was supported by funding from the National Institutes of Health.

## DISCLAIMER

The authors alone are responsible for the views expressed in this issue. They do not necessarily represent the views, decisions or policies of the institutions with which they are affiliated nor any of the funding agencies supporting their work.
